# Heterogeneous Integration of Microelectronics by Self-Assembly

**DOI:** 10.3390/mi17040445

**Published:** 2026-04-03

**Authors:** Yingkun Ma, Karl F. Böhringer

**Affiliations:** Department of Electrical and Computer Engineering, University of Washington, Seattle, WA 98195, USA; yingkunm@uw.edu

As planar CMOS scaling slows, system performance improvements increasingly rely on heterogeneous integration and advanced packaging, shifting cost-per-function pressure onto large-scale assembly and motivating approaches that can deliver both functional diversification (“More-than-Moore”) [1] and function densification beyond historical transistor-scaling trends. Self-assembly—the autonomous organization of components into ordered patterns without human intervention [2]—offers an inherently parallel pathway to microscale integration and can reduce direct-contact handling, which becomes increasingly problematic as device dimensions shrink. The US National Science Foundation identified self-assembly as a particularly promising process for scalable nanomanufacturing [3]. In microscale assembly and packaging, self-assembly is often decomposed into transport, alignment, and permanent attachment steps [4]. A rising question is whether these steps can be engineered to organize millions of building blocks on substrates or 3D architectures with acceptable yield, throughput, speed, and defect rates, since even highly optimized serial placement can become throughput and cost limited at such scales. In practical terms, can self-assembly meet manufacturing metrics such as yield, uniformity, throughput, and defect tolerance at the million-component level?

A useful way to classify self-assembly approaches is by the physical mechanism that provides the driving forces and selectivity. A widely adopted classification includes: (1) folding-based assembly, (2) capillary-force-assisted assembly, (3) shape-matching assembly, (4) magnetic-field-guided assembly, and (5) electric-field-guided assembly. This editorial highlights representative works across these categories, distills their strengths and trade-offs, and frames how scaling toward millions of components shifts the challenge from isolated alignment events to systematic uniformity and yield.

Folding approaches start with planar fabrication, then the use of driving mechanisms to rotate or lift the moving parts out of the plane to assemble 3D structures. This approach is attractive since complex 3D geometries can be produced without 3D printing or manual assembly. Iwase et al. demonstrated sequential batch self-assembly of ferromagnetic microstructures by applying a magnetic field normal to the substrate and programming hinge stiffness. As the magnetic field increased, lower-stiffness hinged elements lifted first, which enables prescribed assembly sequences and the formation of complex 3D shapes [5]. The limitations are also obvious: hinge reliability, stiction during release, and latching mechanisms often affect the structure’s performance. Folding-based approaches provide deterministic geometric transformation rather than statistical capture, making them attractive for 3D microarchitectures but less suited for large-area component transfer. In many realizations, folding works as a self-assembly approach in the sense of autonomous transformation after release, which provides a route to 3D microdevices and packaging features, but it does not directly solve the problem of large-scale parts transfer and placement.

Capillarity defines a geometry-dependent energy landscape whose gradients generate restoring forces and torques. Surface energy minimization is central to many efficient microscale assembly approaches because these restoring forces remain strong at sub-millimeter dimensions. In microscale self-assembly, capillary forces typically arise from liquid menisci that pull components into receptor sites. Capillarity enables passive self-alignment in both part-to-template and part-to-part configurations. In site-specific capillary assembly, a liquid phase is localized to receptor sites through wettability patterning or topographic confinement. Upon contact, components are pinned by the meniscus and subsequently self-aligned as gradients in interfacial energy drive the system toward a lower-energy configuration. Solidification then converts temporary capillary capture into permanent attachment. Detailed experimental studies have quantified this mechanism and its sensitivity to pad geometry and wetting conditions. For example, Samyn et al. demonstrated lithographically defined micrometer-scale adhesive pads that enable accurate capillary alignment [6], while Chang et al. showed how chemical confinement and surface energy contrast define effective capillary traps that drives self-alignment by interfacial-energy minimization [7]. At a large scale, integration yield becomes strongly coupled to receptor layout and interaction statistics rather than alignment accuracy alone. Park et al. analyzed how receptor-site spacing and wetting conditions can suppress undesired multi-site attachment and improve assembly outcomes in dense arrays [8]. For the part-to-part situation, capillarity is also an important stochastic method for forming 2D/3D networks at fluid interfaces, where lateral capillary interactions can be utilized to generate directed structures and flows at interfaces [9]. Overall, stochastic capillary part integration combines massively parallel placement with high intrinsic self-alignment accuracy, and related self-assembly studies have demonstrated the scalability of templated and capillary-guided assembly [10]; however, its scalable yield is ultimately set by the design of receptor wettability and geometry, transport statistics, and also liquid properties. 

Shape-matching self-assembly uses designed receptor-site constraints to encode geometric selectivity. In classic geometric confinement works, assembly parts are transported in a carrier medium and preferentially settle into complementary recessed cavities. This approach is typically coupled with secondary bonding mechanisms to form permanent attachment. Early microsystems demonstrations combined shape-programmed capture with solder-based bonding [11], and Wei et al. combined shape recognition with site-specific solder wetting to enable parallel packaging sequences [12]. Importantly, shape-matching-based self-assembly is not restricted to a liquid medium. Fang et al. demonstrated wafer-level, template-based dry/semidry orienting self-organizing parallel assembly, where parts are agitated and captured by complementary features in air for a dry process or at an air–water interface for a semi-dry process, with capillarity used as an optional refinement step [13]. In summary, shape-matching self-assembly enables receptor-defined selective capture in both liquid and dry environments, and is usually coupled with secondary mechanisms to lock parts into permanent, wafer-level integration.

Magnetic forces are non-contact and can be tuned to be attractive or repulsive, which makes them useful for guiding parts toward desired locations without direct mechanical contact. A representative implementation from Morris et al. demonstrates self-assembly of microscale parts through magnetic guidance coupled with capillary interactions, which uses magnetic forces to improve capture probability and orientation control, and uses capillarity to provide short-range self-alignment and retention once contact is made [14]. Another route is magnetically assisted statistical assembly (MASA). It uses released devices bearing soft-magnetic layers delivered over substrates patterned with cavities that contain perpendicularly magnetized features. After stochastic entry into a cavity, short-range magnetic attraction increases residence time and resists removal by other forces [15]. A recent example demonstrates vertical 3D integration of microchips using magnetic assembly, highlighting magnetic guidance for stacking and heterogeneous integration [16]. Overall, magnetic self-assembly can extend capture range and enable non-contact guidance, but the tradeoff is usually added materials and process complexity. Magnetic guidance increases capture cross-section and residence time but does not inherently guarantee orientation accuracy without complementary short-range alignment forces.

Electric-field-guided self-assembly uses electrokinetic (electrophoresis or electroosmosis) and dielectrophoretic (DEP) force in non-uniform electric fields to move, orient, and localize moving parts without mechanical contact. Because electrode arrays are lithographically defined, electric-field-guided assembly naturally integrates with wafer-scale addressing schemes. An early demonstration of programmable, field-configured assembly showed that mesoscale devices can be transported and placed onto selected receptor electrodes on an addressable substrate, followed by solder reflow to permanently integrate the devices [17]. More recent works combine device geometry and field landscapes to improve orientation selectivity at the chip scale. Lee et al. introduced fin-LED building blocks enabling face-selective DEP assembly, which shows that structural asymmetry can bias DEP force toward a preferred orientation during placement [18]. Chang et al. showed concurrent self-assembly of RGB microLEDs, which highlights that electric-field-based manipulation can be extended to multi-type and parallel assembly [19]. Overall, electric-field-guided self-assembly provides a scalable, non-contact way for transport and placement of parts, but it usually trades this flexibility for electrode complexity and sensitivity to parasitic electrohydrodynamics.

In the More-than-Moore regime, system-level gains increasingly derive from heterogeneous integration and advanced packaging, which shifts cost-per-function pressure onto scalable assembly processes. In this editorial, we focused on engineered self-assembly as a manufacturing and integration strategy, which are the building blocks to autonomously organize into prescribed structures under designed mechanisms and conditions. The scope emphasizes mechanisms relevant to microfabrication and heterogeneous integration, where self-assembly offers parallelism and reduces direct-contact handling as parts size shrink. We discussed the approaches organized by dominant mechanism—folding, capillary, shape matching, magnetic and electric-field-guided mechanisms—highlighting what each method tends to enable in capture, orientation control, and attachment. 

Across mechanisms, scalable microscale self-assembly reduces to the controlled design of interaction potentials under manufacturing variability. The challenge is not alignment in isolation, but statistical reliability across millions of repeated events. The main challenges include wrong orientation, void- and multi-occupancy or aggregation. Another problem is process-window sensitivity because selectivity and driving force depend on parameters that drift in manufacturing. Drifts in surface chemistry, fluid properties, and field conditions can alter the effective energy landscape. For large-scale manufacturing, we can use these criteria to evaluate each self-assembly technique: throughput and delivery uniformity across the entire process, defect rates at the expected rate for target part count, and the programmable ability to assemble multiple part types without excessive complexity. The next phase of microscale self-assembly will likely be defined not by new assembly principles, but by improved control over stochastic transport, energy landscape engineering, and defect tolerance at system scale.
